# Go-CART: an animal-free system for the assessment of CAR T cell function

**DOI:** 10.1186/2051-1426-3-S2-P320

**Published:** 2015-11-04

**Authors:** Pradip Bajgain, Usanarat Anurathapan, Ayumi Watanabe, John Wilson, Sujita Sukumaran, Norihiro Watanabe, Helen Heslop, Cliona Rooney, Malcolm Brenner, Ann Leen, Juan Vera

**Affiliations:** 1 Baylor College of Medicine, Houston, TX, USA; 2 Wilson Wolf Corporation, New Brighton, MN, USA

## 

**Figure 1:**
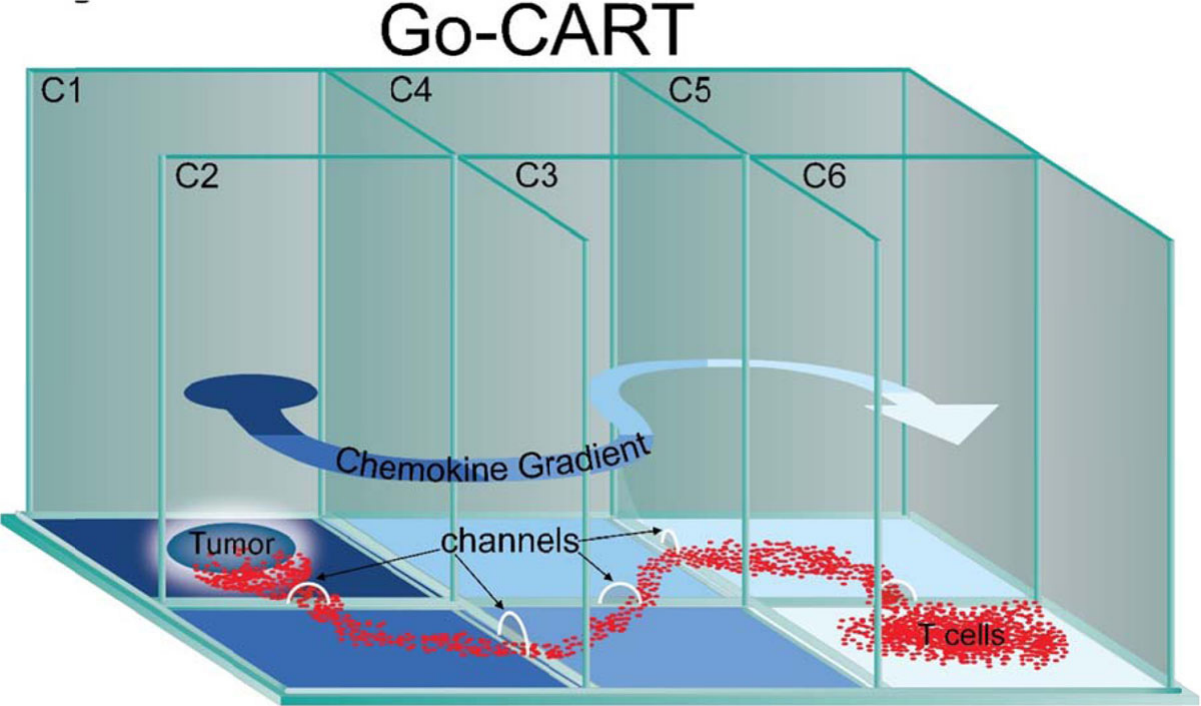


The preclinical development of chimeric antigen receptor (CAR) T cell therapy has been hindered by the inadequacy of current *in vitro* methods to predict T cell performance (i.e. migration, proliferation and anti-tumor activity), which do not recapitulate *in vivo* conditions. For example, most T cell functional assays are performed over a relatively short timeframe (6-18 hrs), and platforms to assess T cell migration and prolonged T cell-tumor cell interactions are very restricted. To overcome these deficits, we developed the Go-CART – an *in vitro*, compartmentalized culture system that allows long-term assessment of multiple biological parameters simultaneously. The Go-CART is a six chambered device (C1-C6) connected by 2mm channels that form an “S” shaped, 11.5cm path, allowing the generation of a chemokine gradient (Figure 1). Thus, T cells and tumor cells can be physically separated, allowing for simultaneous *in vitro* assessment of T cell migration, anti-tumor effects, and persistence. Furthermore, the gas-permeable base of the Go-CART allows for prolonged T cell-tumor cell interaction (>2 weeks) without medium/nutrient replenishment.

To explore whether we could establish a chemokine gradient in the Go-CART, we added 24µg of MCP1 to compartment 1 (C1) and evaluated the chemokine levels in all compartments. After 72 hours, we observed the formation of a gradient that could drive T cell migration (196.79, 78.52, 56.80, 9.79, 2.52 and 0.64ng/ml MCP1, C1-C6, respectively). Next, to assess whether T cell migration could be induced by chemokine-producing tumor cells, we established a 3D tumor model (AlgiMatrix seeded with 1.00E+06 CAPAN1 tumor cells) in C1 and three days later, added 2.00E+07 CAR-PSCA-FFluc-GFP T cells to C6. We monitored T cell migration by periodic bioluminescence imaging and after 5 days, observed an accumulation of T cells at the tumor site (C1) (7.69E+06 and 3.19E+08 p/s/cm^2^/sr, days 1 and 5, respectively). In contrast, T cells failed to localize in the absence of tumor cells. Finally, to assess the utility of the Go-CART in evaluating anti-tumor effects, we established an AlgiMatrix-3D tumor model in C1 with 1.00E+06 CAPAN1-FFLuc-GFP tumor cells and three days later, added 2.00E+07 CAR-PSCA T cells to C6. Under these conditions, we observed a progressive decrease in the tumor signal [3.52E+08 (day 0) and 3.31E+07 (day 15) p/s/cm^2^/sr]. In contrast, in the absence of T cells, the tumor signal progressively increased [2.69E+08 (day 0) to 2.47E+09 (day 15) p/s/cm^2^/sr]. Thus, our results demonstrate that the Go-CART can be used to dynamically assess multiple parameters required for CAR T cell function.

